# Electrical Cardiometry during transition and short-term outcome in very preterm infants: a prospective observational study

**DOI:** 10.1007/s00431-023-05387-1

**Published:** 2024-01-08

**Authors:** C. E. Schwarz, J. M. O’Toole, D. B. Healy, J. Panaviene, V. Livingstone, E. M. Dempsey

**Affiliations:** 1 Department of Paediatrics & Child Health, University College Cork, Cork, Ireland; 2 INFANT Research Centre, University College Cork, Cork, Ireland; 3 Department of Neonatology, Center for Pediatric and Adolescent Medicine, University of Heidelberg, Im Neuenheimer Feld 430, 69120 Heidelberg, Germany

**Keywords:** Bioimpedance, Cardiac index, Electrical Velocimetry, Non-invasive cardiac output, Transition period

## Abstract

The purpose of this study is to evaluate the association of Electrical Cardiometry (EC)-derived cardiac output indexed to weight (CO) and its changes during the first 48 h in relation to adverse short-term outcome in very preterm infants. In this prospective observational study of preterm infants < 32 weeks gestational age (GA), the combined adverse outcome was defined as mortality or abnormal cranial ultrasound (any grade intracranial hemorrhage (ICH) or periventricular leukomalacia) within the first 2 weeks postnatally. Logistic regression models were used to investigate the association between median CO and outcome and mixed-effects models for the time trajectory of CO. In the absence of device-specific thresholds for low or high CO, no thresholds were used in our analysis. Fifty-three infants (median (IQR) GA 29.0 (25.4–30.6) weeks, birthweight 1020 (745–1505) g) were included in the analysis. Median CO was 241 (197–275) mL/kg/min for the adverse outcome and 198 (175–227) mL/kg/min for normal outcome (odds ratio (OR) (95% confidence interval (95% CI)), 1.01 (1.00 to 1.03); *p* = 0.028). After adjustment for GA, the difference was not significant (adjusted OR (95% CI), 1.01 (0.99 to 1.02); *p* = 0.373). CO trajectory did not differ by outcome (*p* = 0.352). A post hoc analysis revealed an association between CO time trajectory and ICH ≥ grade 2.

*          Conclusions*: EC-derived CO estimates within 48 h postnatally were not independently associated with brain injury (any grade) or mortality in the first 14 days of life. CO time trajectory was found to be associated with ICH ≥ grade 2.

**Table:** 

**What is Known:** *• Bioreactance-derived cardiac output indexed to bodyweight (CO) in the transitional period has been associated with adverse short-term outcome in preterm infants.*	
**What is New:** *• Electrical Cardiometry (EC)-derived CO measurements in very preterm infants during the transitional period are not independently associated with adverse outcome (death or ultrasound detected brain damage) within 2 weeks postnatally.* *• In the first 48 h EC-derived CO increases over time and is higher in extremely preterm infants compared to very preterm and differs from previously reported bioreactance-derived CO values.*

**Supplementary Information:**

The online version contains supplementary material available at 10.1007/s00431-023-05387-1.

## Introduction

Non-invasive electrical biosensing technologies such as Electrical Cardiometry (EC) and bioreactance are relatively novel assessment tools for neonates that allow non-invasive continuous objective monitoring of cardiac output (CO). Measured parameters include indices of stroke volume and CO. Over the last decade, both technologies have been increasingly used in various clinical settings in neonatal care despite challenging interpretation due to its limited interchangeability with echocardiography in preterm infants [1–5].

Focusing on the transitional period, Cappelleri et al. were one of the first groups to provide bioreactance data between 6 and 48 h postnatally in a cohort of infants born between 30 + 0 and 34 + 0 weeks gestational age (GA) [6]. They reported a bioreactance-derived CO increment from day 1 to day 2 by 34% [95% confidence interval (95% CI) 21–47%] in line with a stroke volume increment of 29% [16–42%]. Miletin et al. found that for infants < 1250 g with low bioreactance-derived CO on day 1, and CO increasing into the second day, were associated with adverse outcome defined as brain injury or necrotizing enterocolitis [7]. This was interpreted as a reperfusion injury; however, the sample size studied was small (*n* = 39, adverse outcome in 6 participants). Comparative data pertaining to EC are lacking.

Brain injury in preterm infants occurs most frequently within the first days of life, and even low-grade intracranial hemorrhage impacts on long-term outcomes [8–10]. The pathogenesis is multifactorial, but circulatory factors with alterations of cerebral blood flow especially during the transitional period are major contributors [11].

Therefore, we hypothesized that EC-derived CO and its trajectory over time are associated with adverse short-term outcome in very preterm infants. Our aim was to evaluate the association between EC-derived CO, its changes over the first two postnatal days, and the predefined combined adverse outcome of death or cranial ultrasound (crUS) findings attributable to circulatory impairment (any grade intracranial hemorrhage (ICH) or periventricular leukomalacia (PVL)) at 2 weeks of age.

## Methods

### Study design and setting

This prospective observational cohort study was performed in a tertiary level neonatal center (Cork University Maternity Hospital, Ireland). It was approved by the local Research Ethics Committee and registered at clinicaltrials.gov (NCT04538079). Infants were enrolled between November 2019 and May 2021.

### Study population

The inclusion criteria were birth between 23 + 0 weeks and 31 + 6 weeks’ GA with the possibility to commence EC monitoring before 12 h of age. Infants were enrolled following informed parental consent. In cases of twins, only one infant was eligible due to the availability of a single device. Exclusion criteria were declined parental consent, major congenital cardiac defects (except intra-atrial shunts or patent ductus arteriosus (PDA)), severe congenital anomalies, and hydrops fetalis.

### Electrical cardiometry

All participants underwent EC monitoring (ICON, Osypka Medical, Berlin, Germany), which was commenced *as early as possible* but within 12 h of delivery and continued up to the age of 48 h. The detailed technical background was published previously [5]. In short, four neonatal electrodes (iSense, Osypka Medical), placed on the forehead, right side of the neck, left hemithorax at xyphoid level, and left inner thigh, measure changes in electrical conductivity within the thorax which relate to the circulatory system. From this, an estimate of stroke volume and CO is calculated, and these are indexed for birthweight for analysis. The term cardiac output in neonates typically refers to cardiac index, as this is the term used for CO indexed to bodyweight. Hereafter, CO refers to cardiac index. Data processing for EC outputs is detailed in the supplementary material.

### Cranial ultrasound

All participants underwent routine crUS performed by the attending radiologists as part of standard care (unit standard first crUS within 3 days of life, second between 7 and 10th day of life). The Philips HD-11XE with a C8-5 transducer (convex curved array 8–5 MHz probe, Philips, Netherlands) or GE Logiq P9 with an 8C transducer (micro convex curved array 4–8 MHz probe, GE Healthcare, USA) was used. Radiologists were unaware of the EC values. Papile et al. classification of brain injury was used [12]. The highest grade of ICH diagnosed and the diagnosis of PVL within the first 2 weeks was documented.

### Clinical, neonatal, and echocardiographic data

Clinical and neonatal data (including GA, birthweight, Apgar scores, CRIB-II score), data on respiratory support (including surfactant use and pneumothorax), and mortality were extracted from electronic charts. Functional echocardiography was performed at least once during the monitoring period (first 48 h) to ensure normal cardiac anatomy, and for PDA evaluation, PDA was classified according to its flow pattern: pulsatile non-restrictive flow considered a hemodynamically significant PDA (Vivid-I, 10S-RS sector array transducer, frequency range 4.5–11.5 MHz or Vivid-E9, 12S sector phased array transducer, 4.0–12.0 MHz) (both GE Healthcare, USA). Echocardiography was performed by neonatologists trained in neonatal echocardiography (EMD, CES, Neidin Bussmann). This institution’s general approach to PDA management is conservative, and none of the infants received treatment for PDA during the initial 48-h monitoring period.

### Two-week outcome

Infants were divided into normal and adverse outcome groups. The adverse outcome was defined as a combined outcome including mortality and/or abnormal crUS (any grade ICH or periventricular leukomalacia) within the first 2 weeks of life. For a post hoc sensitivity analysis, infants with low-grade ICH (grade 1) were considered to have a normal outcome.

### Statistical analysis and sample size

The planned target sample size was 100 participants to allow for multiple comparisons.

Categorical variables were described using frequencies and percentages and continuous variables using medians and interquartile ranges (IQRs). For comparisons between outcome groups (adverse, normal), the Mann–Whitney *U* test was used for continuous variables and the *χ*^2^ test or Fisher’s exact test (in the case of small expected counts) was used for categorical variables. Logistic regression models were used to investigate the association between median CO across the first 48 h of life and outcome, both unadjusted and adjusted for GA group. GA was dichotomized into either extremely preterm (< 28 weeks) or very preterm (≥ 28 weeks and < 32 weeks) for the analysis. The Hosmer–Lemeshow goodness of fit test was used to determine the fit of the logistic regression models with *p* < 0.05 indicating lack of fit. Prior to performing the multivariable logistic regression analysis, multicollinearity among the independent variables was tested using the variance inflation factor (VIF) and VIF < 10 was considered acceptable. Linear mixed-effects models were fitted to the data to investigate if the CO trajectory over time differed by outcome group. The initial model included intercept, time (postnatal age), group, the interaction of time by group and GA group as fixed effects and intercept and time as random effects. The dependent variable was CO. Statistical significance for the fixed effects was determined using Satterthwaite’s method and 95% CI for fixed effects were computed using a bootstrap method with 1000 iterations. Model selection was based on a step-down model building approach, starting with a complex model (initial model), followed by backward elimination of random effects and then backward elimination of fixed effects (final model).

The mixed model was implemented in *R* (version 4.0, *R* Foundation for Statistical Computing, Vienna, Austria) using *lme4* (version 1.1) and *lmerTest* (version 3.1) libraries. All other analyses were performed using IBM SPSS Statistics (version 28, IBM Corp, Armonk, NY, USA). All tests were two-sided and a *p* value < 0.05 was considered to be statistically significant.

## Results

### Participant demographics

**Fig. 1:**
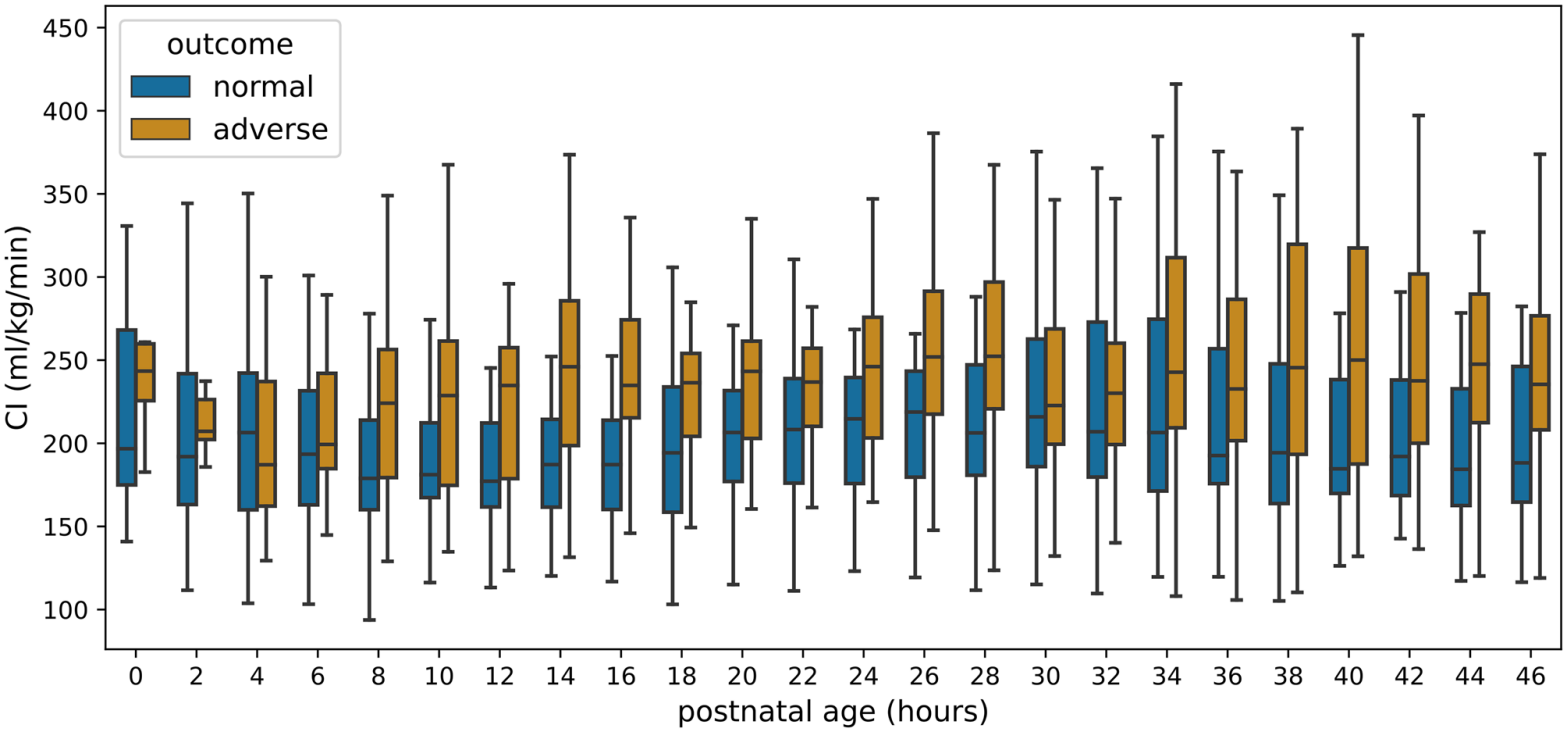
Two-hourly mean cardiac output indexed to bodyweight (CO) within first 48 h of age according to predefined adverse or normal outcome (colored in online supplement)

Fifty-six infants were enrolled, of whom 53 infants were included in the analysis. Excluded infants had severe pulmonary stenosis (*n* = 1), a genetic syndrome (*n* = 1), and insufficient EC recording time < 12 h (*n* = 1) (Fig. 1S). Neonatal and demographic variables are summarized in Table 1 (see Table 1S for additional information). EC monitoring was commenced at a median (IQR) age of 3.2 (1.9 to 5.7) h for a duration of 44.3 (41.3 to 46.0) h.

**Table 1:** Baseline characteristics of study cohort overall and by outcome group

**Variables**	Study cohort (*n* = 53)*n* (%)*	**Outcome group**		***p* value**
		Adverse outcome (*n* = 21) *n* (%)*	Normal outcome (*n* = 32) *n* (%)*	
Gestational age [weeks]: median (IQR)	29.0 (25.4 to 30.6)	25.1 (24.2 to 30.5)	30.1 (28.0 to 30.6)	0.005^a^
Birth weight [g]: median (IQR)	1020 (745 to 1505)	770 (645 to 1405)	1190 (891 to 1600)	0.011^a^
Sex [female]	29 (55)	13 (62)	16 (50)	0.394^b^
Multiples	9 (17)	4 (19)	5 (16)	1.0^c^
Intrauterine growth restriction	11 (21)	3 (14)	8 (25)	0.494^c^
Antenatal steroids (completed)	36 (68)	12 (57)	24 (75)	0.173^b^
Antenatal MgSO_4_ Not available	45 (87) 1	16 (76) 0	29 (94) 1	0.104^c^
Mode of delivery [caesarean]	35 (66)	11 (52)	24 (75)	0.089^b^
Apgar score min 5: median (IQR) Not available	8 (6 to 8) 1	7 (6 to 8) 0	8 (6 to 9) 1	0.127^a^
Clinical risk index for babies II score	7 (3 to 13)	14 (4 to 15)	6 (3 to 10)	0.004^a^
Any cardiovascular support within 48 h Type of cardiovascular support Volume-therapy	6 (11) 6	6 (29) 6	0	0.002^b^
Inotropes	5	5		
Hydrocortisone	4	4		

*Unless otherwise stated

^a^From Mann–Whitney *U* test

^b^From *χ*^2^ tests

^c^From Fisher’s exact test

### CO by outcome group

The adverse outcome occurred in 21 (40%) of the infants (death (*n* = 4, of whom 3 had abnormal crUS) and/or abnormal crUS (any grade ICH (*n* = 19) or PVL (*n* = 1))). This group had lower GA and birthweight, a higher rate of hsPDA, and received more respiratory and circulatory support within the first 48 h.

Median (IQR) CO within the first 48 h of age was 241 (197 to 275) mL/kg/min for infants in the adverse outcome group and 198 (175 to 227) mL/kg/min for those in the normal group (odds ratio (OR) (95% CI), 1.01 (1.00 to 1.03); *p* = 0.028). After adjustment for GA, this difference was no longer significant (adjusted OR (95% CI), 1.01 (0.99 to 1.02; *p* = 0.373)) (Fig. 2S). The Hosmer–Lemeshow goodness of fit test indicated best fit with GA groups (compared to GA as continuous variable) and a VIF < 10 indicated no relevant multicollinearity.

Figure 1 illustrates the course of CO over the monitoring period. Linear mixed modeling was used to analyze differences between groups. Backwards and forwards selection resulted in the same model. The final model (Table 2) includes intercept and time (postnatal age) as random effects and intercept, time, and GA group as fixed effects. This model indicates that coming from a baseline (intercept) CO of 185 (95% CI 168 to 201) mL/kg/min, CO increased over time (+ 17 mL/kg/min per day; 95% CI 8 to 26). Furthermore, CO was higher in extremely compared with very preterm infants (+ 39 mL/kg/min; 95% CI 17 to 62) (Fig. 3S).

**Table 2:** Estimates (95% CI) of the linear mixed-effects model for cardiac output (indexed to bodyweight)

	**Initial model**			**Intermediate model**			**Final model**		
	Coefficient	(95% CI)	*p* value	Coefficient	(95% CI)	*p* value	Coefficient	(95% CI)	*p* value
Intercept	185	(167 to 203)	< 0.001	182	(165 to 199)	< 0.001	185	(168 to 201)	< 0.001
Time [days]	13	(2 to 25)	0.032	17	(8 to 27)	< 0.001	17	(8 to 26)	< 0.001
Extremely preterm GA group (ref: very preterm)	32	(8 to 57)	0.021	32	(9 to 60)	0.021	39	(17 to 62)	0.002
Adverse outcome (ref: normal)	9	(− 21 to 36)	0.575	15	(− 14 to 40)	0.275			
Outcome × time [days]	9	(− 10 to 27)	0.352						

*GA* gestational age

A post hoc analysis with re-classification of ICH grade 1 (*n* = 9) as a normal outcome was subsequently performed. This revealed a significantly greater increase in CI over time in infants with ICH ≥ grade 2 (+ 35 mL/kg/min/day compared to + 12 mL/kg/min per day, see Table 1S and Fig. 4S).

## Discussion

This is the first study of very preterm infants to evaluate relationships between CO derived by EC and relevant short-term outcomes. In our study, the adverse outcome occurred in 40% of the participants and was more common in the extremely preterm subgroup. We found that after adjusting for GA there was no association between CO and outcome. We identified an increase in CO over time and higher CO in extremely preterm infants. Our model suggested that differences in CO trajectories between the outcome groups were explainable by GA.

Miletin et al. identified a significant association between adverse outcome (ICH ≥ grade 2 or necrotizing enterocolitis ≥ grade 2A) and CO measured using bioreactance within the first 6 to 48 h postnatally [7]. While the study included 39 infants, only six infants in that study had the predefined adverse outcome. As compared to EC, bioreactance uses four dual-electrodes applied to the thorax and analyzes phase shift between an applied current and the measured voltage signal [1]. There are other important differences between these studies: Monitoring was commenced earlier in our study (44 (77.8%) of the participants < 6 h of age); the definition of adverse outcome differs, the endpoint of assessment time differs, and most importantly, our study adjusts for GA, a major predictive factor for ICH in preterm infants [13]. In contrast to the primary analysis, the post hoc analysis indicated that infants in the revised adverse outcome group (excluding grade 1 ICH) had a significantly higher increase in CO over the monitoring period compared to those in the revised normal group (including grade 1 ICH). This supports the previous finding described by Miletin et al. suggesting a higher CO at day 2 to adverse outcome and adds evidence for the potential underlying pathophysiology of reperfusion injury.

Another important finding is in the comparison between EC-derived and bioreactance-derived CO values: EC was higher by a factor of approximately two compared to values reported using bioreactance. A direct comparison between bioreactance- and EC-derived CO parameters is lacking. However, this finding is consistent with previous work comparing both technologies separately to echocardiography. In relation to CO, EC was noted to overestimate echocardiography, while bioreactance was found to underestimate echocardiography CO measures [1, 3, 14]. This underlines the need for technology-specific reference values. In line with Miletin et al., bioreactance-derived CO was found to change with postnatal age within the transitional period in a cohort of more mature preterm infants. Comparable to recent studies using bioreactance [6, 7, 15], our model also shows that EC-derived CO increased during the transitional period. Normative values for EC-derived CO in the transitional period are limited. For the first week of life in more mature preterm infants (mean (SD) GA 31 (3.2) weeks), Boet et al. reported absolute values for EC-derived CO (not indexed to bodyweight). [16]. Hu et al. published normative values for EC-derived absolute CO for > 72 h postnatally. Based on data from 11 infants < 28 weeks, 12 infants between 29 and 30 weeks, and 31 infants between 31 and 32 weeks GA to be 0.23 (0.03), 0.29 (0.06), and 0.35 (0.07) mL/min, respectively [17]. Due to the differences in the cohorts and the timing of measurements, comparability to our study is lacking and further research is required. In the absence of device-specific thresholds for low or high CO in the transitional period, no threshold-driven analysis was performed in our study.

Low CO in the transitional period, measured by echocardiography with superior vena cava flow as a surrogate marker to adjust for neonatal shunts, was found to be associated with adverse outcome, including ICH [18–20]. However, the multifactorial nature of death and brain injury in preterm infants implies end-organ blood flow as one of several contributing factors [21]. Real-time continuous monitoring and analysis of CO may help to identify underlying pathologies and to guide the need for clinical evaluation and management. However, as both EC and bioreactance have been found to be non-interchangeable with echocardiography in preterm infants [2–4], caution is advised in clinical interpretation of CO values measured by these technologies. Furthermore, fetal shunts which commonly affect a preterm infants’ circulation need to be considered. For both technologies, accuracy may be improved after the transitional period when these shunts may have decreased. As such, the role of EC should not replace echocardiography but might have utility for monitoring trends. While the usefulness of bioreactance to monitor trends was found to be limited [14], data for EC [22] are lacking.

Associations between other non-invasive circulatory monitoring technologies and adverse outcomes have also been identified. Pulse oximetry-derived perfusion index has been used in preterm infants for identifying low systemic blood flow [23]. Low values and decreased variability within the first day of life were found to be associated with adverse outcome in extremely premature infants [24]. Cerebral tissue oxygenation obtained by near-infrared spectroscopy in the transitional period has been found to be associated with adverse short- and long-term outcome in preterm infants [25, 26] and a large multinational randomized trial is currently evaluating clinical incorporation and its potential effects on outcome [27].

### Limitations

This is a single-center study and recruitment was stopped before reaching the original target sample size. Recruitment was below our estimation for several reasons, including inability to enroll multiple simultaneous participants and interruptions due to local COVID-19 restrictions (see Fig. 1S). Therefore, multifactorial and subgroup analysis is limited [13, 28].

Whereas normal crUS is predictive for a normal neuromotor and cognitive outcome in preterm infants [29], the ability of abnormal crUS to predict impaired outcome is limited. ICH severity is associated with mortality, disability, or cerebral palsy and potentially results in hydrocephalus [30, 31]. The predefined primary outcome included all grades of ICH. For ICH grade 1–2, the probability of abnormal neuromotor and cognitive outcome is only 9%, while for grade 3 this increases to 26% [29]. Hemorrhagic parenchymal infarction (grade 4) predicts abnormal neurodevelopmental outcome with a positive predictive value of 47% [32]. However, even low-grade ICH was found to affect long-term outcome [10, 33–35]. Our primary endpoint was at 2 weeks, which includes the vast majority of ICH [8], but may not capture all PVL, some of which may only become apparent on ultrasound after this time [32, 36].

## Conclusion

This study revealed that CO measured using EC in very preterm infants in the first 2 postnatal days is not independently associated with adverse outcome. CO increased over time and was found to be higher in extremely preterm compared with very preterm infants. In a post hoc analysis of infants with ICH grade ≥ 2, the increase over time was more pronounced in the adverse outcome group. EC-derived data seems to vary significantly compared to bioreactance-derived CO. In the absence of optimal normograms for EC-derived CO within the first hours of life in extremely preterm infants, our study provides valuable insights to CO and its change over time in the transitional period. Further evaluation is required before routine application of the technology in the clinical neonatal setting could be recommended.

### Supplementary Information

Below is the link to the electronic supplementary material.

Supplementary file1 (DOCX 2047 KB)

## Data Availability

Additional de-identified data will be made accessible on reasonable requests. Requests should be directed to the corresponding author.

## Abbreviations

95% CI: 95% Confidence interval
CO: Cardiac output (indexed to bodyweight)
crUS: Cranial ultrasound
EC: Electrical Cardiometry
GA: Gestational age
IQR: Interquartile range
OR: Odds ratio
(hs)PDA: (Hemodynamically significant) patent ductus arteriosus
ICH: Intracranial hemorrhage
PVL: Periventricular leukomalacia
