# Supplementary material for: Electrical Cardiometry during transition and short-term outcome in very preterm infants: a prospective observational study
Source: Eur J Pediatr. 2024 Jan 8;183(4):1629–36. doi: 10.1007/s00431-023-05387-1 (PMC11001706; doi:10.1007/s00431-023-05387-1)
Supplement: Supplementary file 1 — Supplementary file1 (DOCX 2047 KB) [file 431_2023_5387_MOESM1_ESM.docx]

**Electrical Cardiometry (detailed data processing)**

The signal quality index (SQI) in EC was used to extract reliable measurements in beat-to-beat data. SQI estimates the percentage of reliable measurements within the previous 10 beats.[5] Therefore, only data with increasing SQI or the maximum of 100% were used for further analysis.

Cardiac output indexed to bodyweight (CO) data was then averaged over a 1-minute period with an overlap of 50% (30 seconds). To ensure sufficient data in this average, the 1-minute segment was split into 2 30-second segments. If the 30 second segment has <6 reliable beats, then this was rejected from further analysis. Infants with <12 h of valid CO data were excluded.

Next, the data was low-pass filtered to create a slowly-varying trend component.[6] The filtering was implemented using an infinite impulse response (IIR) filter, designed with a Butterworth filter of order 11 with a cut-off frequency of 1/3600 Hz. A forward–backwards pass was used to ensure zero phase. To maintain a uniform sampling rate, missing data was estimated using piece-wise cubic interpolation. After filtering, the interpolated segments were removed. The trend component was down sampled to 1/600 Hz, which equates to 1 sample per 10 minutes.

**Table 1S:** Additional Baseline characteristics of study cohort overall and by outcome group

| **Variables** |  | **Outcome group** | |  |
| --- | --- | --- | --- | --- |
|  | **Study cohort**  **(n=53)**  **n(%)*** | **Adverse Outcome**  **(n=21)**  **n(%)*** | **Normal Outcome**  **(n=32)**  **n(%)*** | ***p-***  **value** |
| PDA within 48 h  PDA closed  Non-hsPDA  HsPDA  Not assessed | 13 (25)  25 (48)  14 (27)  1 | 3 (15)  8 (40)  9 (45)  1 | 10 (31)  17 (53)  5 (16)  0 | .058‡ |
| Highest mode of respiratory support within 48 h  Non  Continuous positive airway pressure  High flow nasal canula  Invasive intermitted positive pressure ventilation  High frequency oscillation ventilation  Inhaled nitric oxide | 3 (6)  21 (40)  3 (6)  21 (40)  5 (9)  3 (6) | 3 (14)  4 (19)  0  9 (43)  5 (24)  3 (14) | 0  17 (53)  3 (9)  12 (38)  0  0 | <.001§  .057§ |
| Surfactant therapy | 33 (62) | 15 (71) | 18 (56) | .265‡ |
| Pneumothorax within first 48 h | 2 (4) | 2 (10) | 0 | .152§ |
| Blood transfusion within 48 h | 9 (17) | 7 (33) | 2 (6) | .021§ |
| Early onset sepsis (culture pos.) | 1 (2) | 1 (5) | 0 | .396§ |

*unless otherwise stated; (hs)PDA (hemodynamically significant) patent ductus arteriosus,

‡ from χ2 tests;§ from Fisher’s exact test.

**Table 2S.** Estimates (95% CI) of the linear mixed-effect model for cardiac output indexed to body weight (adverse outcome excluding, normal outcome including IVH grade 1)

|  | coefficient | (95% CI) | p-value |
| --- | --- | --- | --- |
| Intercept | 189 | (173 to 204) | <.001 |
| Time [days] | 12 | (2 to 21) | .027 |
| Extremely preterm GA group (ref: very preterm) | 28 | (-3 to 58) | .068 |
| Adverse Outcome x time [days] | 23 | (2 to 43) | .039 |
| Adverse Outcome (ref: normal) | 4 | (-34 to 43) | .575 |

GA: gestational age


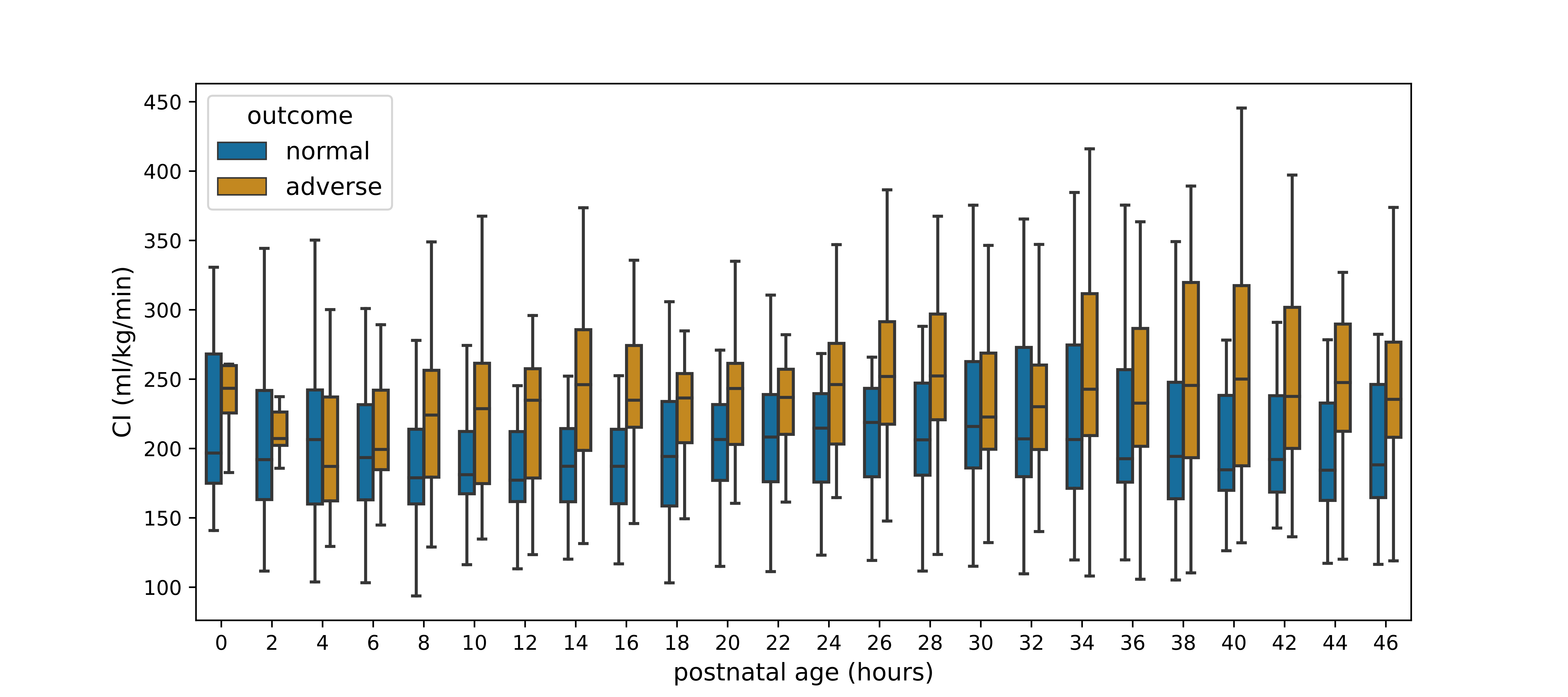


**Figure 1** **(online supplement colored version)** Two-hourly mean cardiac output indexed to bodyweight (CO) within first 48 hours of age according to predefined adverse or normal outcome


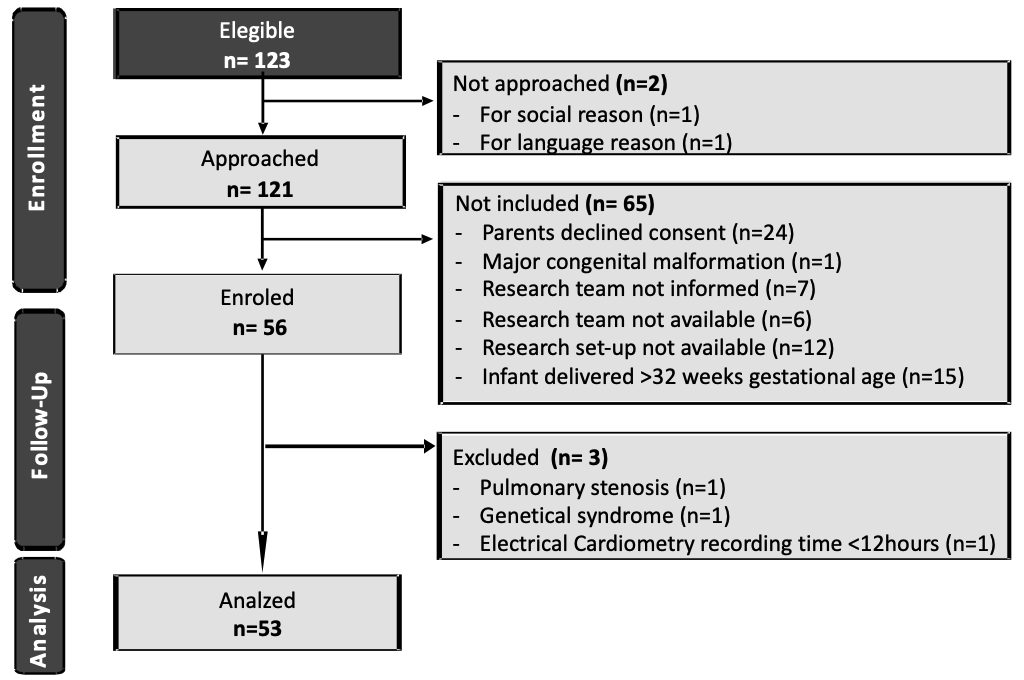


**Figure 1S** **(online supplement)** STROBE Flow chart

**
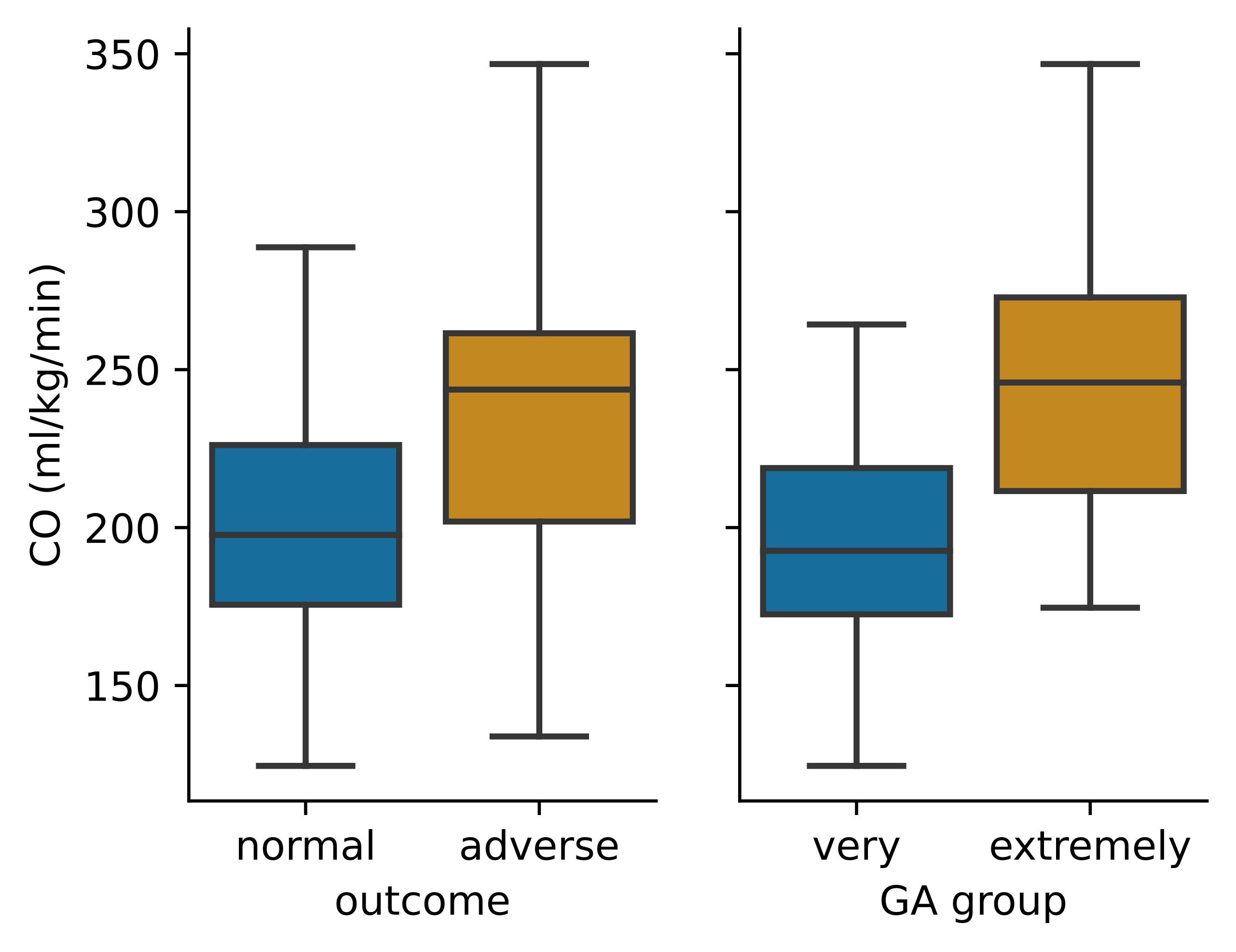
**

**Figure 2S (online supplement)** Box-plots for median cardiac output indexed to bodyweight, (CO) for 48 h of age grouped according to predefined adverse outcomes vs grouped according to gestational age (GA; very (≥28weeks) vs extremely (<28weeks) preterm)


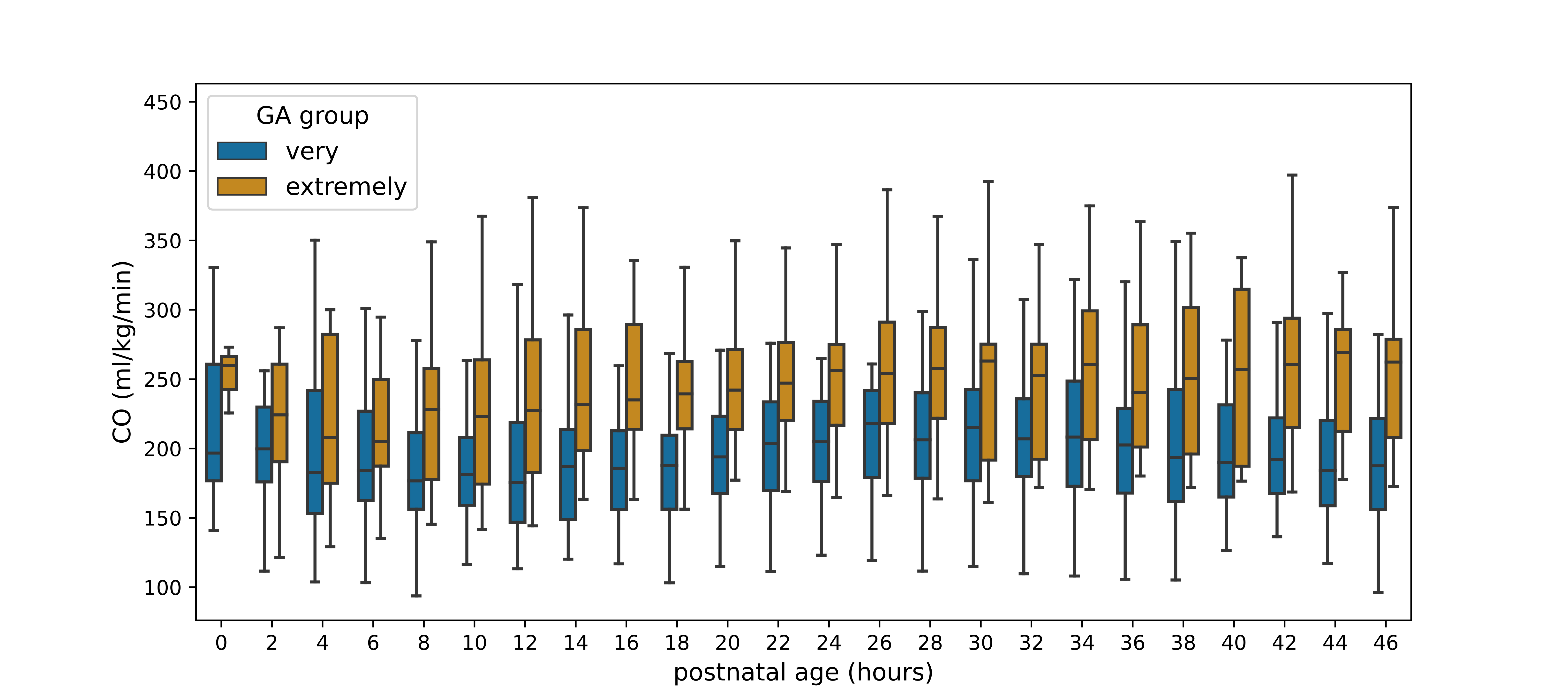


**Figure 3S (online supplement)**: Box-plots for two-hourly median cardiac output indexed to body weight (CO) within first 48 hours of age according to gestational age (GA) group (very (≥28weeks) vs extremely (<28weeks) preterm)

**
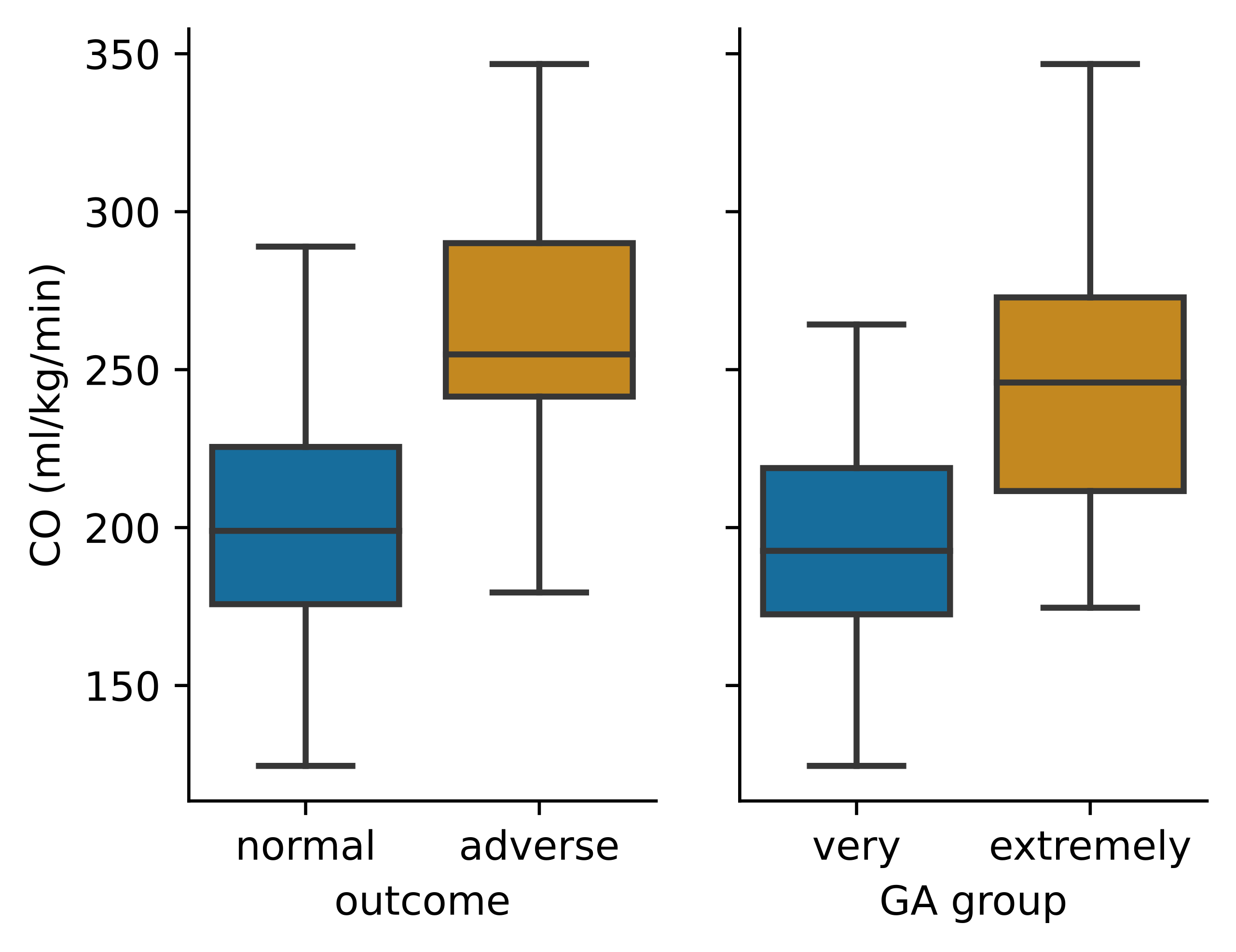
**

**Figure 4S (online supplement)** Box-plots for median cardiac output indexed to body weight (CO) for 48 h of age grouped according to post-hoc defined adverse outcomes (ICH grade 1 classified as normal outcome) vs grouped according to gestational age (GA; very (≥28weeks) vs extremely (<28weeks) preterm)
